# Prognostic Significance of Different Ventricular Ectopic Burdens During Submaximal Exercise in Asymptomatic UK Biobank Subjects

**DOI:** 10.1161/CIRCULATIONAHA.123.064633

**Published:** 2023-10-19

**Authors:** Stefan van Duijvenboden, Julia Ramírez, Michele Orini, Nay Aung, Steffen E. Petersen, Aiden Doherty, Andrew Tinker, Patricia B. Munroe, Pier D. Lambiase

**Affiliations:** 1 Institute of Cardiovascular Science, University College London, United Kingdom (S.v.D., M.O., P.D.L.).; 2 William Harvey Research Institute, Barts and The London School of Medicine and Dentistry, Queen Mary University of London, United Kingdom (S.v.D., J.R., N.A., S.E.P., A.T., P.B.M.).; 3 Nuffield Department of Population Health, University of Oxford, United Kingdom (S.v.D., A.D.).; 4 Aragon Institute of Engineering Research, University of Zaragoza, Spain and Centro de Investigación Biomédica en Red – Bioingeniería, Biomateriales y Nanomedicina, Spain (J.R.).; 5 Barts Heart Centre, St Bartholomew’s Hospital, London, United Kingdom (M.O., N.A., S.E.P. P.D.L.).; 6 NIHR Barts Biomedical Research Centre, Barts and The London School of Medicine and Dentistry, Queen Mary University of London, United Kingdom (N.A., S.E.P., A.T., P.B.M.).

**Keywords:** cardiovascular diseases, electrocardiography, exercise, mortality, ventricular premature complexes

## Abstract

**BACKGROUND:**

The consequences of exercise-induced premature ventricular contractions (PVCs) in asymptomatic individuals remain unclear. This study aimed to assess the association between PVC burdens during submaximal exercise and major adverse cardiovascular events (MI/HF/LTVA: myocardial infarction [MI], heart failure [HF], and life-threatening ventricular arrhythmia [LTVA]), and all-cause mortality. Additional end points were MI, LTVA, HF, and cardiovascular mortality.

**METHODS:**

A neural network was developed to count PVCs from ECGs recorded during exercise (6 minutes) and recovery (1 minute) in 48 315 asymptomatic participants from UK Biobank. Associations were estimated using multivariable Cox proportional hazard models. Explorative studies were conducted in subgroups with cardiovascular magnetic resonance imaging data (n=6290) and NT-proBNP (N-terminal Pro-B-type natriuretic peptide) levels (n=4607) to examine whether PVC burden was associated with subclinical cardiomyopathy.

**RESULTS:**

Mean age was 56.8±8.2 years; 51.1% of the participants were female; and median follow-up was 12.6 years. Low PVC counts during exercise and recovery were both associated with MI/HF/LTVA risk, independently of clinical factors: adjusted hazard ratio (HR), 1.2 (1–5 exercise PVCs, *P*<0.001) and HR, 1.3 (1–5 recovery PVCs, *P*<0.001). Risks were higher with increasing PVC count: HR, 1.8 (>20 exercise PVCs, *P*<0.001) and HR, 1.6 (>5 recovery PVCs, *P*<0.001). A similar trend was observed for all-cause mortality, although associations were only significant for high PVC burdens: HRs, 1.6 (>20 exercise PVCs, *P*<0.001) and 1.5 (>5 recovery PVCs, *P*<0.001). Complex PVC rhythms were associated with higher risk compared with PVC count alone. PVCs were also associated with incident HF, LTVA, and cardiovascular mortality, but not MI. In the explorative studies, high PVC burden was associated with larger left ventricular volumes, lower ejection fraction, and higher levels of NT-proBNP compared with participants without PVCs.

**CONCLUSIONS:**

In this cohort of middle-aged and older adults, PVC count during submaximal exercise and recovery were both associated with MI/HF/LTVA, all-cause mortality, HF, LTVAs, and cardiovascular mortality, independent of clinical and exercise test factors, indicating an incremental increase in risk as PVC count rises. Complex PVC rhythms were associated with higher risk compared with PVC count alone. Underlying mechanisms may include the presence of subclinical cardiomyopathy.

**Clinical Perspective**

**What Is New?**

- This study assessed the risk for a wide range of premature ventricular complex (PVC) counts, during submaximal exercise and recovery, in the largest population-based cohort study to date with exercise data, comprising 48 400 asymptomatic individuals with a mean age of 56.8±8.2 years from the UK Biobank study.
- Results show incremental increases in risk between PVC burden during submaximal exercise and major adverse cardiovascular events, heart failure, and ventricular arrhythmias, independently of clinical risk factors, during a median follow-up period of over >10 years in this population.

**What Are the Clinical Implications?**

- Widespread use of wearable cardiac rhythm monitors, many of which are used during exercise, has enabled detection of PVCs in asymptomatic individuals at larger scale and is expected to result in more consultation requests from concerned patients whose monitors detect PVCs.
- Consequently, clinicians need to be aware of the potential prognostic significance of high PVC burden during and after exercise in middle-aged and older adults without known cardiovascular disease.

Exercise testing poses a dynamic physiological stress on the heart that may unmask underlying cardiac anomalies not evident at rest and is commonly ordered to guide risk assessment in low- or intermediate-risk individuals.^1^ Premature ventricular complexes (PVCs) are commonly observed during exercise testing (prevalence ≈7%).^2^ The prognostic implications of high PVC burden are well-recognized in patients with structural heart disease^3–7^; however, the implications for asymptomatic individuals remain incompletely understood. As highlighted recently, the increased availability of wearable ECG-monitoring devices has significantly improved the identification of asymptomatic participants with PVCs, emphasizing the need to better understand the association between PVCs and cardiovascular risk.^8^

The prognostic implications of low and intermediate PVC counts are unknown, and little is known about the specific risks carried by different PVC rhythms (eg, couplets, triplets, and bigeminy). A very limited number of studies have investigated the prognostic implications of PVCs on exercise in asymptomatic individuals.^8–14^ The latest evidence suggests that high-grade PVCs (frequent or complex PVCs) during recovery after exercise are associated with a 1.7 times higher long-term cardiovascular mortality risk, independent of established clinical risk factors, whereas no significant association was found for PVCs during exercise in 5486 individuals of which 311 died of cardiovascular disease.^8^ However, the quality of the evidence is limited because study cohorts are small, usually not population based, and, as highlighted previously, there is a lack of uniformity among the definitions used to designate frequent PVCs.^15^ In addition, little is known about the association of PVCs with other important cardiac outcomes, including myocardial infarction (MI), life-threatening ventricular arrhythmia (LTVA), and heart failure (HF). We hypothesized that the risk associated with PVCs increases with frequency of PVCs and their pattern.

In the present study, we investigated PVCs observed during submaximal exercise in 48 502 asymptomatic individuals without known cardiovascular disease with >10 years follow-up from the UK Biobank study to address the following hypotheses: (1) PVC burden during submaximal exercise (n=0, 1–5, 6–10, 11–20, >20 beats) and recovery (n=0, 1–5, >5) determines the clinical adjusted risk for major adverse cardiovascular events (MI/HF/LTVA) and all-cause mortality. (2) PVC burden is associated with MI, LTVA, and HF, and cardiovascular mortality. (3) Levels of risk differ according to the different PVC patterns.

## METHODS

### UK Biobank

The UK Biobank is a large population cohort established between 2006 and 2010, with even numbers of men and women 40 to 69 years of age on recruitment from 21 assessment centers across England, Wales, and Scotland. The study has extensive baseline and follow-up clinical, biochemical, and outcome measures and approval from the North West Multi-Centre Research Ethics Committee. All participants provided informed consent at the time of enrollment for their data to be linked to the health record systems. This research has been conducted using the UK Biobank Resource under application numbers 8256 and 2964.

### Exercise Test

From the full UK Biobank cohort, 95 154 participants consented to participate in a submaximal exercise test between 2009 and 2013. The test used cycle ergometry on a stationary bike (eBike, Firmware v1.7) in conjunction with a single lead (lead I) electrocardiograph device (CAM-USB 6.5, Cardiosoft v6.51, sample rate 500 Hz). The exercise protocol consisted of a 15-s pretest resting phase, followed by graded activity (6 minutes) and a recovery period with hands remaining on the handlebars while remaining still and silent (1 minute, no cool-down period). According to protocol, participants were risk-stratified into groups, and those who were allowed to exercise were categorized to perform a test at either 35% or 50% of the maximum predicted workload depending on their risk profile.^16^ A submaximal workload allowed testing to be conducted safely across participants with a wide fitness range, including those not normally considered for exercise testing. The predicted absolute maximum workload was calculated according to a formula, which includes age, height, weight, resting heart rate, and sex. We excluded participants who prematurely terminated their test due to (chest) discomfort.

### ECG Analysis

Because we did not have access to a method to detect and count PVCs, we developed, trained, and validated a deep-learning model to detect PVCs from raw single-lead ECG recordings from the exercise cohort in the UK Biobank study. ECGs were first bandpass filtered (0.5–45 Hz), downsampled to 250 Hz, and then split in nonoverlapping segments of 2048 time samples (≈8.2 s) for computational efficiency. A one-dimensional convolutional neural network was trained on the ECG segments to estimate the probabilities for each time sample in the segment that it belonged to: (1) a normal (narrow) QRS complex, (2) a PVC QRS morphology, and (3) none of the above (Figure S1). The network was implemented using the Keras framework with a TensorFlow (Google) backend and Python (v3.8.5).^17^ The main architecture was composed of 2 one-dimensional convolutional layers connected to a dropout, dense, and classification layer. Postprocessing was applied to rejoin segments and to filter the estimated probabilities to improve localization and classification of QRS complexes in the ECG.

**Figure 1.**
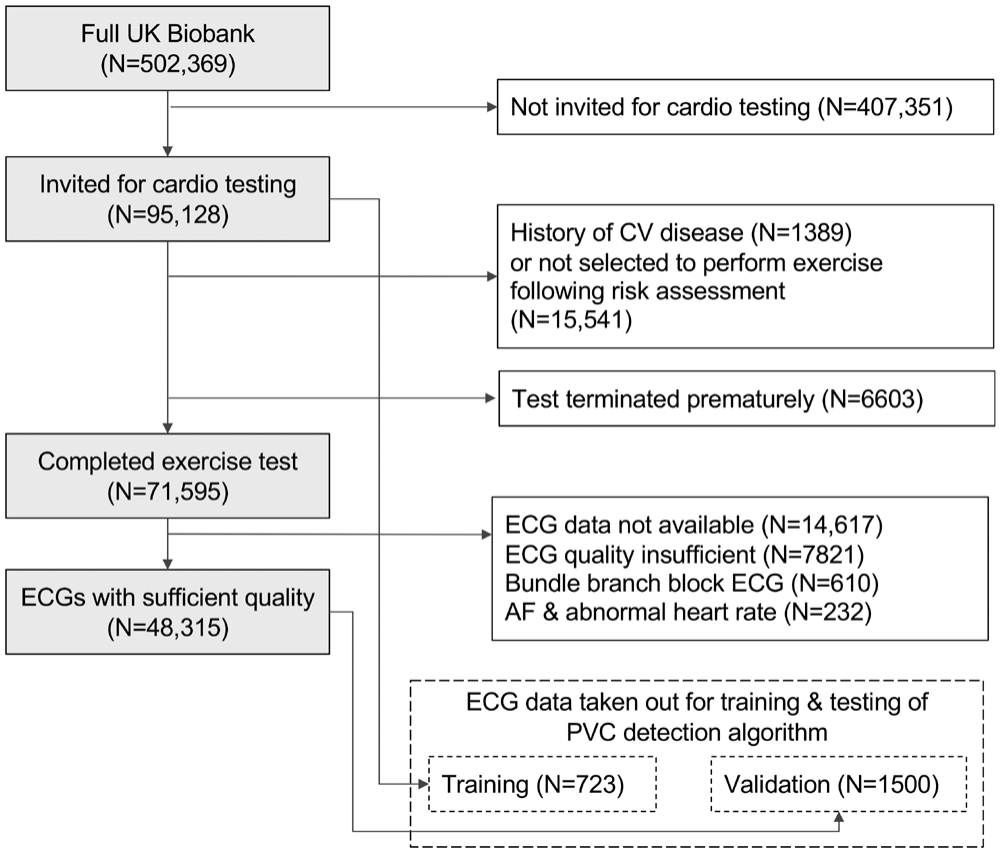
**Study exclusion diagram.** History of cardiovascular (CV) disease was identified using self-reported and hospital episode data (Table S1). AF indicates atrial fibrillation; and PVC, premature ventricular contraction.

The network was trained on 41 025 ECG segments from 723 participants who had at least 1 PVC during the exercise test identified after visual inspection of exercise ECGs from 1142 participants with abnormal RR intervals from data previously derived by our group.^18^ The performance algorithm was tested in a dataset of 79 257 ECG segments from 1500 participants that were randomly selected from the remaining ECGs in UK Biobank from participants without cardiovascular disease (Figure 1). All ECGs in training and testing sets were manually reviewed by an expert (SVD) to localize all QRS complexes and to label them as normal beats or PVC. A PVC was defined as a QRS-T complex with QRS duration ≥0.12 s not preceded by at least 0.12 s by a P wave.

It is important to note that the distribution of PVC count was highly skewed (Figure S2) with excessive zero PVC counts in most participants (79.7% and 90.1% having 0 PVCs for exercise and recovery, respectively). We therefore evaluated the algorithm’s performance to accurately predict 5 predefined categories (0, 1–5, 6–10, 11–20, and >20 PVCs), which were chosen on the basis of clinical utility. Satisfactory performance was achieved: in the validation dataset, the overall accuracy was 98.7%, and sensitivity and positive predictive values ranged between 89.2% to 100.0% and 94.3% to 99.8%, respectively, across the PVC count categories (Table S1). The algorithm was used to analyze all remaining ECGs in our dataset that were not part of training or a validation set.

To compare our findings with the recently published work from Refaat et al,^8^ we reviewed ECGs with PVCs for high-grade PVCs using the same criteria: frequent (>10 per minute), multifocal, >2 consecutive PVCs in a row (including ventricular tachycardia), or R-on-T type. R-on-T and multifocal PVCs were manually confirmed by reviewing all ECGs with short PVC coupling interval (<400 ms) or low correlation among PVC beats (correlation coefficient <0.75).

### Ascertainment of Study End Points

All UK Biobank participants consented to be followed up through linkage to their health-related records and the UK death register.^19^ Participants with known cardiovascular disease before the exercise test, identified by using either health-recorded or self-reported in the baseline questionnaire, were excluded (codes are provided in Table S2). The primary study end point was major adverse cardiovascular events (MI/HF/LTVA), defined as either hospitalization or death due to MI, HF, or LTVA. Cases were identified using relevant *International Classification of Diseases, 10th Revision* (ICD-10) or OPCS Classification of Interventions and Procedures version 4 (OPSC4) codes in the health-related records or death register (Table S3). The secondary end point was all-cause mortality. We also performed 2 additional analyses: (1) to evaluate the associations with MI, LTVA, and HF separately and (2) the association with cardiovascular death. The follow-up period was determined by the first appearance of ICD-10 or OPSC4 codes in either health record or death register data. Participants who did not experience an event were censored at death or the end of the follow-up period (November 30, 2022).

### Statistical Analysis

Due to its occasional nature, the distribution of PVC count is highly skewed, with a majority of participants having zero PVC count. We therefore categorized PVC count into frequency groups to capture the varying levels of PVC burden (0 PVCs, 1–5 PVCs, 6–10 PVCs, 11–20 PVCs, and >20 PVCs for exercise, and 0, 1–5, >5 PVCs for recovery). Using these systematic interval groupings, we aimed to create reasonably balanced risk groups while preserving the interpretability of the results. Descriptive statistics are presented as mean±SD for continuous variables or frequency (percentage) for categorical variables. Baseline clinical and exercise ECG characteristics of the groups with PVCs were statistically compared with the group with no PVCs. Bonferroni correction was applied for multiple testing (n=4 for exercise PVCs and n=2 for recovery PVCs). We used Wilcoxon rank-sum tests for continuous variables and the χ^2^ or Fisher exact test for categorical variables, whichever was appropriate. We constructed survival probability curves for MI/HF/LTVA and all-cause mortality stratified for PVC count. The association between PVC count and study end points was investigated using multivariable-adjusted Cox proportional hazards regression. A minimally adjusted analysis used sex, age, and the number of heart beats during exercise or recovery. By including the number of heart beats (either during exercise or recovery), we estimated the risk carried by PVCs independently from their frequency with respect to the total number of heart beats during exercise or recovery. In a second model we addressed potential sources of confounding by further adjusting for clinical variables (hypertension, type 2 diabetes, low-density lipoprotein cholesterol and high-density lipoprotein cholesterol, triglycerides, smoking, body mass index) and (exercise) ECG variables (QTc interval, QRS duration, ST-segment depression during exercise of >0.1 mV, and heart rate increase during exercise (for exercise PVCs) or decrease during recovery (for recovery PVCs). Hypertension was defined as systolic blood pressure of ≥140 mm Hg or diastolic blood pressure of ≥90 mm Hg measured on the day of assessment^20^ or a previous a diagnosis of hypertension (details provided in Table S4). We estimated 95% CIs for each group (including the reference [0 PVCs] group) that corresponded to the amount of information underlying each group.^21^ Inspection of Schoenfeld residuals revealed a possible violation of the proportional hazards assumption by sex. To avoid potential bias, we stratified Cox models by gender.

Missing variables (smoking status [0.4%], low- and high-density lipoprotein cholesterol [8.2% and 13.2%, respectively], triglycerides [8.2%], body mass index [<0.01%], QRS duration [0.9%], QTc interval [4.6%], ST depression [0.02%]) were imputed using the multiple imputation by chained equations approach, with 5 imputed datasets and 10 iterations.^22^ For each variable we specified a predictive mean matching model, and we used all baseline variables to inform the imputation. Imputations were found acceptable by comparison of plots of the distribution of recorded and imputed values for all measurements. Cox regression models were estimated separately for each imputed dataset and then pooled together to obtain one overall set of estimates. *P*<0.05 was considered statistically significant. Statistical analyses were performed in R version 4.0.2.^23^

### Sensitivity Analyses

We performed sensitivity analyses to examine the potential confounding influence of high PVC burden at rest and 2 major noncardiovascular diseases (chronic obstructive pulmonary disease and chronic kidney disease [CKD]). High PVC burden at rest was defined as ≥1 PVC recorded during the available 15-s (preexercise) resting period. This information was added as a binary covariate to the fully adjusted Cox models. Chronic obstructive pulmonary disease was identified using recommended definitions published previously by UK Biobank.^24^ CKD was defined as CKD stages 3 to 5 (estimated glomerular filtration rates <60 mL·min·^–1^1.73 m^–2^), analogous to previous work in UK Biobank^25^ where estimated glomerular filtration rate values were calculated using the Chronic Kidney Disease Epidemiology Collaboration formula.^26^ The contributing effect was then investigated by repeating the main analysis after excluding participants with prevalent chronic obstructive pulmonary disease or CKD.

### Explorative Analysis: Association Between PVC Burden and Markers of (Subclinical) Cardiomyopathy

In an explorative subgroup analysis, we examined whether subclinical cardiomyopathy could be a potential contributing factor to the associations between high PVC burden and cardiovascular outcomes. In one analysis, we evaluated the association between high PVC burden and functional and structural parameters derived from cardiovascular magnetic resonance imaging. This study was conducted in a subgroup of participants who had imaging data derived during the UK Biobank imaging follow-up study. Prognostically important markers of cardiomyopathy (left ventricular mass, ejection fraction, end-diastolic volume, stroke volume, and myocardial native T1) were derived using a fully automated quality-controlled image analysis pipeline previously developed and validated in UK Biobank.^27,28^ In a second analysis, we evaluated the association with serum levels of NT-proBNP (N-terminal Pro-B-type natriuretic peptide, an established biomarker of HF and ventricular dysfunction) measured at baseline.^29^ Measurements below the protein’s lower limit of detection were substituted by limit of detection/√2.^30^ Models in both cardiovascular magnetic resonance and brain natriuretic peptide analyses were adjusted using the same covariates as the Cox regression analysis. In addition, results were also adjusted for the time interval between exercise test and imaging study for cardiovascular magnetic resonance parameters.

## RESULTS

After the exclusions for reasons highlighted in Figure 1, 48 315 participants without known cardiovascular disease and with complete ECGs were included in the study. The study population comprised a balanced set of middle-aged men and women (51.1% female, mean age 56.8±8.2 years). Tables 1 and 2 summarize the number of participants in each PVC count category during exercise and recovery, respectively. Participants with higher PVC counts were older, more likely to be male, to be hypertensive, and to have diabetes. They were also more likely to show ST-segment depression (>0.1 mV). After a median follow-up period of 12.6 years (interquartile range, 3.5 months), there were 2175 (4.5%) MI/HF/LTVA events (hospitalization or death) and 2441 (5.1%) participants died of any cause.

**Table 1.**
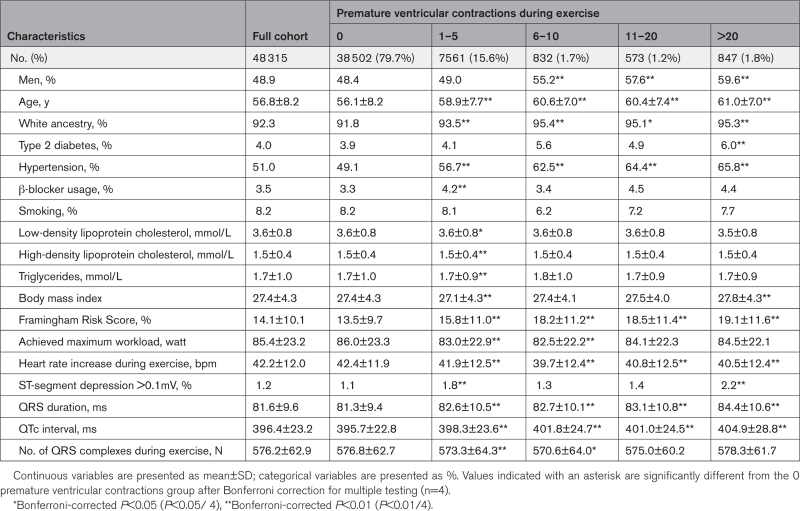
Baseline Clinical and Stress Test Characteristics During Exercise

**Table 2.**
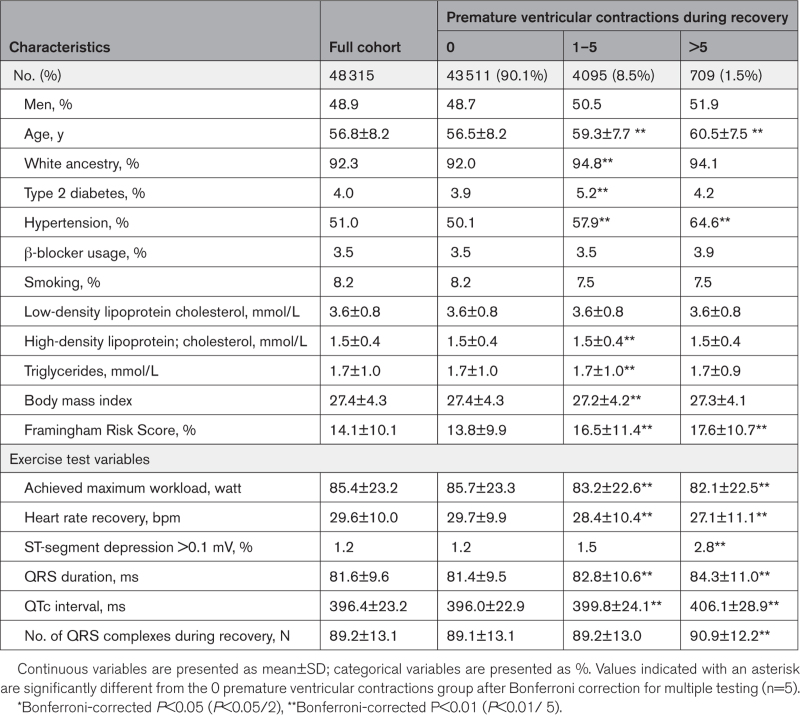
Baseline Clinical and Stress Test Characteristics During Recovery

### PVCs During Exercise

**Figure 2.**
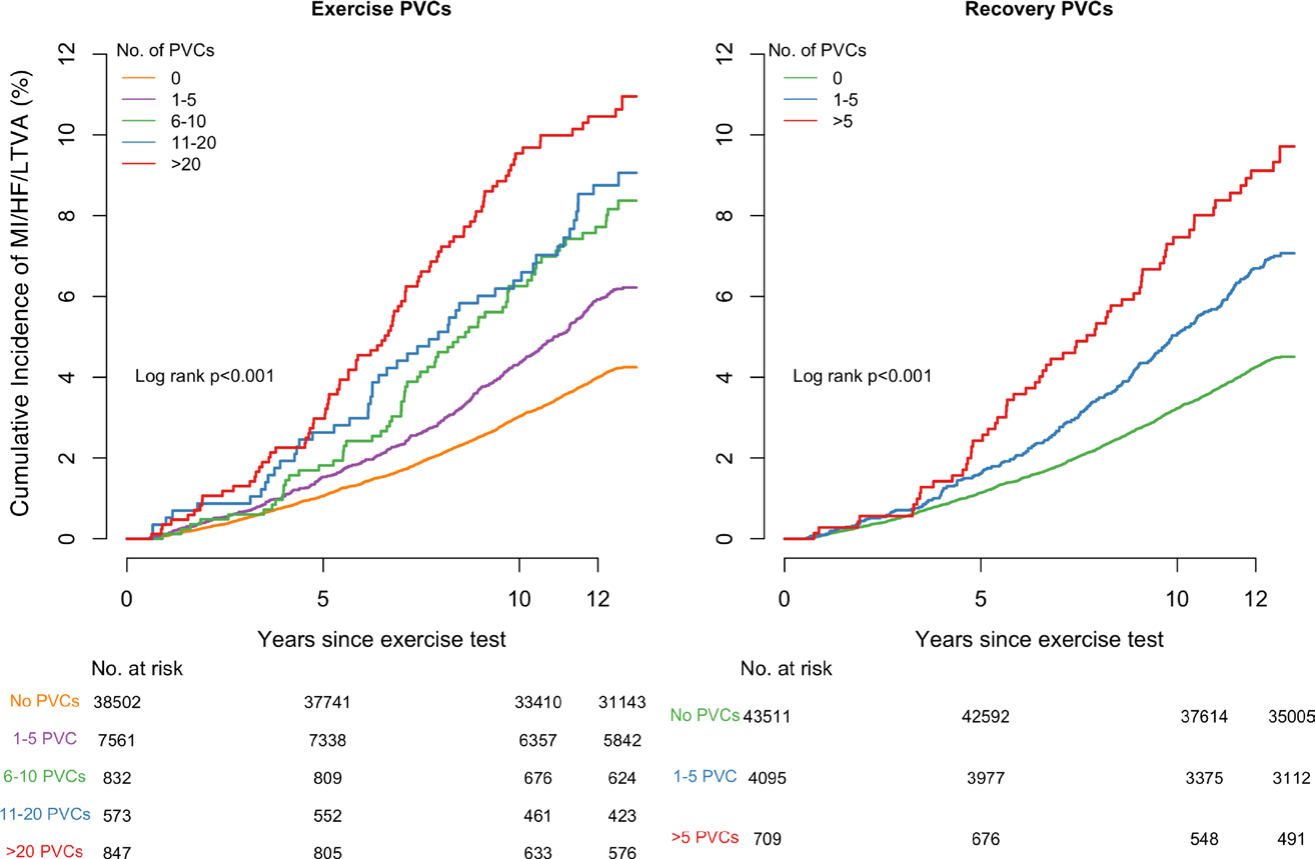
**Cumulative incidence curves of MI/HF/LTVA events according to different PVC counts.** HF indicates heart failure; LVTA, life-threatening ventricular arrhythmia; MI, myocardial infarction; and PVC, premature ventricular contraction.

**Figure 3.**
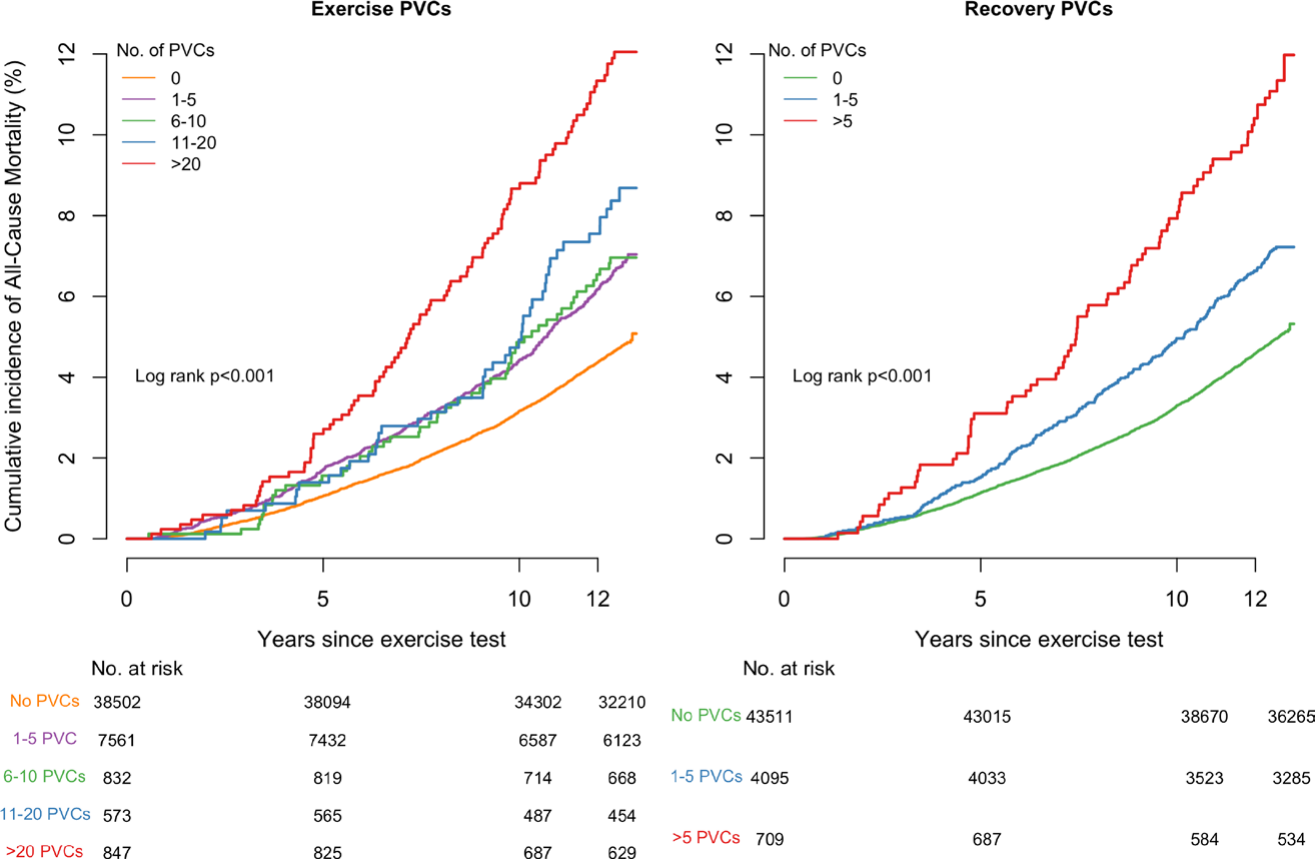
**Cumulative incidence curves of all-cause mortality events according to different PVC counts.** PVC indicates premature ventricular contraction.

The incidence of MI/HF/LTVA events and all-cause mortality were significantly higher in participants with PVCs during exercise and increased with PVC count. In the group without PVCs, the incidence was 4.0% compared with 5.8% in the group with 1 to 5 PVCs (*P*<0.001). Among participants with PVCs, the incidence further increased to 10.2% (>20 PVCs) and was significantly higher than in the group with 1 to 5 PVCs (*P*<0.001). A similar trend was observed for all-cause mortality: the incidence increased from 4.6% (0 PVCs) to 6.5% (1–5 PVCs, *P*<0.001). Among participants with PVCs, the incidence further increased to 11.4% (>20 PVCs, *P*<0.001). The unadjusted Kaplan-Meier curves showed decreasing survival rates for both MI/HF/LTVA and all-cause mortality with increasing PVC counts (*P*<0.001; Figures 2 and 3).

**Figure 4.**
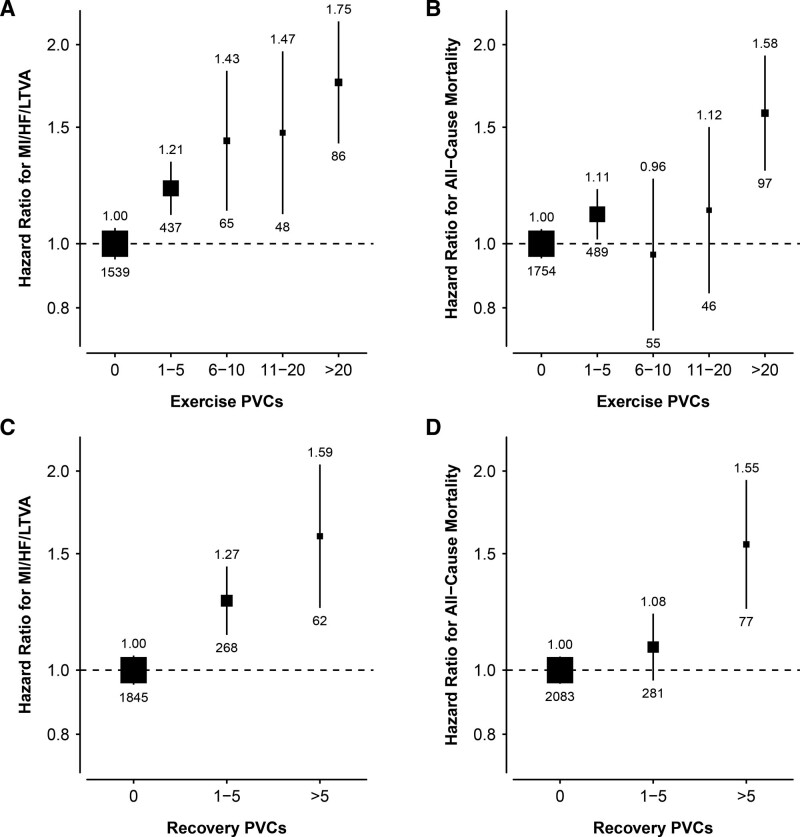
**Adjusted hazard ratios of MI/HF/LTVA events and all-cause mortality according to PVC count.** Hazard ratios were adjusted for clinical variables: age, sex, diabetes, hypertension, β-blocker medication, smoking, Low-density lipoprotein cholesterol and high-density lipoprotein cholesterol, triglycerides, body mass index, QRS duration, QTc interval, ST depression (>0.1 mV), and number of heartbeats during exercise and heart rate exercise (for exercise PVCs), or number of heartbeats during recovery and heart rate recovery (for recovery PVCs). The reference category is 0 PVCs. Sizes of the boxes are proportional to the inverse of the variance of the log-transformed hazard ratios. Vertical lines represent 95% CIs. Numbers shown above and below each box are the hazard ratio and number of events, respectively. HF indicates heart failure; LVTA, life-threatening ventricular arrhythmia; MI, myocardial infarction; and PVC, premature ventricular contraction.

After adjusting for age, sex, and the number of heartbeats during exercise, all PVC counts were significantly associated with MI/HF/LTVA and remained significant after further adjusting for clinical factors (Figure 4A, Table S5). The risk increased with PVC count with HRs ranging from: 1.2 (95% CI, 1.1–1.3, *P*<0.001) for 1 to 5 PVCs to 1.8 (95% CI, 1.4–2.2, *P*<0.001) for >20 PVCs. For all-cause mortality, the strongest association was found for >20 PVCs (fully adjusted HR, 1.6 [95% CI, 1.3–1.9], *P*<0.001; Figure 4B). We also found a weak, but significant, association for 1 to 5 PVCs (fully adjusted HR, 1.1 [95% CI, 1.0–1.2], *P*=0.022), but associations were not significant for the other (higher) PVC burdens (6–10 and 11–20 PVCs, Figure 4B, Table S5). In the further adjusted models, myocardial ischemia (ST depression) was not significantly associated with MI/HF/LTVA or all-cause mortality (HR, 1.2 [95% CI, 0.8-1.8], *P*=0.443), and HR, 1.4 [95% CI, 1.0–1.9], *P*=0.092), respectively).

### PVCs During Recovery From Exercise

The MI/HF/LTVA incidence was significantly higher in the group with PVCs during recovery: 4.2% (0 PVCs) versus 6.5% (1–5 PVCs, *P*<0.001). Among participants with PVCs, the incidence was higher in those with >5 PVCs compared with 1 to 5 PVCs (8.7% [>5 PVCs], *P*=0.040). A similar trend was observed for all-cause mortality: the incidence increased to 6.9% (1–5 PVCs, *P*<0.001) and 10.9% (>5 PVCs, *P*<0.001). The Kaplan-Meier curves showed increasing event rates for both MI/HF/LTVA and all-cause mortality with increasing PVC counts (*P*<0.001; Figures 2 and 3).

After adjusting for age, sex, and the number of heartbeats during recovery, PVC count was significantly associated with MI/HF/LTVA and results remained similar after further adjusting for clinical factors (adjusted HRs, 1.3 [95% CI, 1.1–1.4], *P*<0.001, 1–5 PVCs; and 1.6 [95% CI, 1.2–2.0], *P*<0.001) for >5 PVCs (Figure 4C, Table S6). Only the highest PVC count (>5 PVCs) was associated with all-cause mortality (HR, 1.5 [95% CI, 1.2–1.9], *P*<0.001; Figure 4D).

### Combining PVCs During Exercise and Recovery

A majority of the participants with repetitive PVCs during exercise also had PVCs during recovery: 435 (51%) from all participants with >20 PVCs during exercise also had >5 PVCs during recovery. This group comprised 61% of the participants with >5 PVCs during recovery. After adjusting for clinical risk factors, the combination of >20 PVCs during exercise and >5 PVCs during recovery was associated with both MI/HF/LTVA (HR, 1.7 [95% CI, 1.2–2.3], *P*<0.001) and all-cause mortality (HR, 1.6 [95% CI, 1.2–2.2], *P*<0.001).

### Association With Additional Study End Points

The number of MI, LTVA, and HF events were 1378 (2.9%), 307 (0.6%), and 838 (1.7%), respectively. There were 502 (1.0%) cardiovascular deaths. PVCs during exercise and recovery were both associated with incident LTVAs, HF, and cardiovascular mortality, but not with MI (Tables S7 and S8). The highest risk was observed for the highest PVC counts: For exercise, the fully adjusted HRs of >20 PVCs were as follows: 3.6 (95% CI, 2.3–5.7, *P*<0.001), 2.8 (95% CI, 2.1–3.7, *P*<0.001), and 2.8 (95% CI, 1.9–4.0, *P*<0.001), for LTVA, HF, and cardiovascular mortality, respectively. Similarly, the HRs of >5 recovery PVCs were: 2.9 (95% CI, 1.8–4.9, *P*<0.001), 2.4 (95% CI, 1.7–3.4, *P*<0.001), and 2.3 (95% CI, 1.5–3.5, *P*<0.001) for LTVA, HF, and cardiovascular mortality, respectively.

### Prognostic Value of High-Grade and Complex PVC Rhythms

To compare our data with the recently published work from Refaat et al,^8^ we identified high-grade PVCs in 1560 (3.2%) and 727 (1.5%) participants, during exercise and recovery, respectively. High-grade PVCs were associated with MI/HF/LTVA independent of clinical and ECG factors (HR, 1.7 [1.4–2.0], p<0.001; HR, 1.7 [1.3–2.1], *P*<0.001, for exercise and recovery, respectively; Figure S3) and all-cause mortality (HR, 1.3 [95% CI, 1.1–1.5], *P*=0.003; HR, 1.3 [95% CI, 1.0–1.7], *P*=0.028). Both markers were also associated with cardiovascular mortality (HR, 2.3 [95% CI, 1.7–3.1], *P*<0.001, and HR, 2.0 [95% CI, 1.3–3.2], *P*=0.001; Table S9). Complex PVC rhythms (couplets, triplets, and bigeminy) were all associated with higher risk for MI/HF/LTVA compared with PVC count alone with fully adjusted HRs ranging between 2.4 (single PVC couplet) and 7.1 (≥1 episode with ≥3 consecutive PVCs; Figure S3).

### Sensitivity Analyses

Results obtained after adjusting for high resting PVC burden (n=1437, 3.0%) or excluding participants with prevalent chronic obstructive pulmonary disease (n=466; 1.0%) or CKD (n=1374; 3.1%) were similar to the main analyses for both exercise and recovery PVCs (Tables S10 and S11).

### Exploring Associations Between Exercise-Induced PVC Burden and Markers of Cardiac Structure and Function

Cardiovascular magnetic resonance measurements were available for 6290 (13.0%) participants from our study cohort and were taken on an average of 6.4±2.3 years after the exercise test. The group with high PVC burden had larger left ventricular end-diastolic and systolic volumes and lower ejection fractions than participants without PVCs (Tables S12 and S13). After adjusting for clinical and exercise ECG variables, high exercise PVC burden remained significantly associated with larger volumes (≈14 mL and ≈11 mL, *P*<0.001, for end-diastolic and systolic volume, respectively) and lower ejection fraction (–3%, *P*<0.001; Table S14). Findings were similar for high PVC burden during recovery, although the effects were smaller (Table S14). No significant associations were found for native myocardial T1 and left ventricular mass and stroke volume. Serum levels of NT-proBNP measured at the time of the exercise test were available in 4607 (9.5%) participants and were higher in participants with a high PVC burden compared with those without PVCs (Tables S15 and S16). Considering that 1 unit increase in Normalized Protein eXpression equates a doubling of protein concentration, high PVC burden was associated with a ≈10% (*P*=0.008) and ≈13% (*P*<0.001) increase in NT-proBNP protein concentration, for exercise and recovery, respectively (Table 3).

**Table 3.**
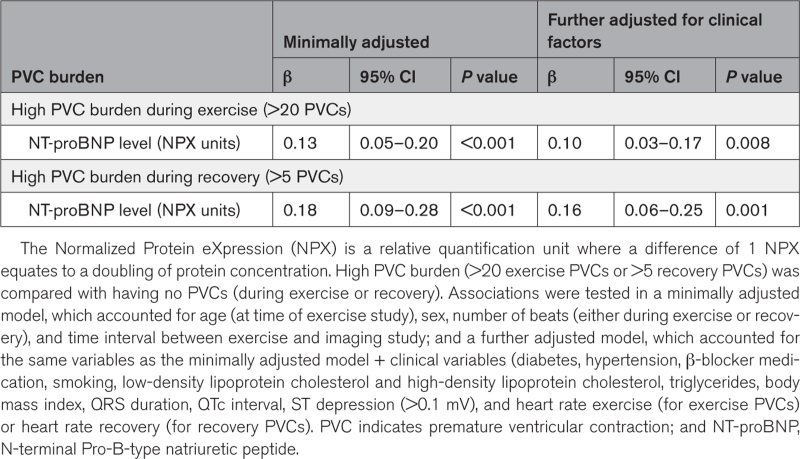
Multivariable Regression Results for the Association Between High PVC Burden and Serum NT-proBNP Levels

## DISCUSSION

To our knowledge, this is the largest population-based cohort study to date to investigate the prognostic value of exercise-induced PVCs in asymptomatic individuals without known cardiovascular disease during submaximal exercise. The main important findings are as follows: (1) low PVC counts (1–5 PVCs during exercise or recovery) were already significantly associated with MI/HF/LTVA, independent of clinical risk factors; (2) PVC count during submaximal exercise and recovery are both associated with MI/HF/LTVA, mortality, and cardiovascular death; (3) there is an incremental risk of MI/HF/LTVA for increasing PVC count; and (4) complex PVCs rhythms are associated with higher risk compared with PVC count alone. This has important implications in the assessment of asymptomatic individuals, in particular, with the advent of wearable ECG technologies worn by otherwise healthy individuals during daily activity and exercise prompting cardiological review, but keeping in mind that the study group had a mean age of 56.8 ±8.2 years when the data on exercise were collected. Also, the exercise performed here had limited intensity.

 Refaat et al^8^ recently investigated the prognostic value of PVCs during exercise versus recovery in 5486 asymptomatic patients not suspected of having heart disease from the Lipid Research Clinics study. Compared with previous studies, they used a more inclusive definition of “high-grade PVCs” (>10 PVCs during a 60-s interval, >2 consecutive PVCs including ventricular tachycardia, R-on-T PVCs, or multiform PVCs). Their data showed that high-grade PVCs during recovery were significantly associated with higher risk of cardiovascular mortality (adjusted HR, 1.7 [1.1–2.6]; *P*=0.02), but no associations were found for high-grade PVCs during exercise. Furthermore, high-grade PVCs (neither during exercise nor recovery) were not associated with all-cause mortality after adjusting for clinical variables. Our data from >48 000 participants of UK Biobank only partially support these findings. In a submaximal exercise test, we found that high-grade PVCs during exercise and recovery were both associated with cardiovascular mortality, MI/HF/LTVA, and all-cause mortality, using the same definition for high-grade PVCs and included similar clinical and exercise parameters as covariates. It is possible that conflicting differences in baseline characteristics between both populations (eg, higher proportion of patients with hyperlipidemia and smokers compared with our cohort) could explain these differences. Sensitivity analysis from Refaat et al^8^ showed no link between PVC risk and hyperlipidemia, but other work investigating PVC load and aortic stiffness did find an association between the number of PVCs in 24-hour ambulatory monitoring.^31^ Another important difference between our and Refaat’s work is the exercise protocol. The activity performed by UK Biobank participants in this study was limited to either 35% or 50% of the maximum predicted workload, whereas participants in Refaat’s study^8^ exercised at maximal Bruce protocol. As a result, the cause of PVCs observed in our and their study may be different. For example, plasma catecholamine levels have been linked with exercise intensity^32,33^ and may mediate PVCs. It is possible that participants in Refaat et al^8^ experienced higher catecholaminergic levels by exercising at maximum workload and that their exercise PVCs are more likely to reflect a physiological response to exercise compared with UK Biobank. However, this explanation may not be fully compatible with the incremental risk observed in this study, which suggests that the risk for MI/HF/LTVA increases with PVC count.

To our knowledge, this is the first study to document an incremental risk for exercise-induced PVCs in asymptomatic individuals without cardiovascular disease. In this study, uniquely, we did not use one cutoff threshold for PVC burden but evaluated the risk for a wide range of PVC counts, during exercise and recovery. Little is known about the risk associated with low and intermediate PVC counts. Two previous studies have investigated the prognostic value of infrequent PVCs: Jouven et al^9^ observed infrequent PVCs (<10% of all ventricular depolarizations, no consecutive PVCs) in 8.5% and 7.3% of a middle-aged male population during exercise and recovery, respectively. They found that cardiovascular and total mortality rates were higher for infrequent PVCs with respect to participants without PVCs; however, they only performed formal association studies for frequent PVCs (≥10% of all ventricular depolarizations). In the second study, Morshedi-Meibodi et al^13^ investigated infrequent and frequent PVCs during submaximal exercise (< and ≥0.22/min of exercise) and found that both types of PVC were significantly associated with mortality in 2885 participants from the Framingham Offspring Study during 15 years of follow-up (adjusted HR, 1.9 [1.2–2.8], and 1.7 [1.2–2.5], for infrequent and frequent PVCs, respectively). In our cohort, this cutoff value translates to between 1 and 2 PVCs during exercise. Both 1 to 5 PVC counts were not associated with all-cause mortality in this work.

### Pathophysiological Rationale

Previous studies in both patients and asymptomatic participants have suggested that PVCs during recovery carry a worse prognosis for (cardiovascular) mortality compared with PVCs during exercise.^6,8^ This has fueled the hypothesis that the prognostic value of exercise-induced PVCs might be linked to attenuated vagal reactivation during recovery, perhaps by reduced suppression of PVCs. Our data do support this hypothesis but indicate at least equal prognostic value for PVCs during submaximal exercise. PVCs during exercise may reflect increased norepinephrine levels during exercise in high-risk individuals, for example, because they had to produce more effort during exercise than their healthier counterparts who may have more cardiopulmonary reserve and skeletal muscle efficiency. The mechanisms of how PVCs during exercise and recovery are related to cardiovascular events remain to be further explored. Our explorative study suggested that high PVC burden is associated with higher brain natriuretic peptide levels measured at the same day as the exercise test, and we also observed differences in cardiac function and structure during follow-up imaging, which may both support existence of subclinical cardiomyopathy. However, PVCs observed during submaximal exercise may have a different cause than PVCs observed during maximal stress testing. The associations between PVC count and LTVA and HF implicate the existence of an arrhythmic substrate (ie, fibrosis or ischemia), which enables reentry and reduces cardiac function precipitating these events. The development of PVCs during exercise and recovery indicates the presence of this underlying substrate with PVCs arising due to the effects of subclinical ischemia (reflected by ST depression on exercise) and adrenergic stress causing triggered ectopic activity that could be due to early afterdepolarizations typically occurring in conditions of catecholamines, tissue injury, altered electrolytes, hypoxia, or acidosis.^34^

Increased PVCs during exercise may also reflect the presence of subclinical pathological myocardial substrate or ischemia. Although ST depression was not associated with MI/HF/LTVA or mortality, it carried a higher risk for LVTA and HF. Ischemia can promote optimal conditions for ventricular arrhythmias and impair left ventricular function through multiple mechanisms, and it is well recognized to increase myocardial refractoriness to promote wave break and polymorphic ventricular tachycardia.^35,36^ However, the fact that single-lead ST measurements with submaximal exercise could result in underestimation of ischemia burden is a potential limitation in this study. Using 2 bipolar leads (V5 and V5R) and a maximal exercise test, frequent exercise-induced PVCs and ischemia were previously independent predictors of cardiovascular mortality with similar HRs in asymptomatic men.^9^ In that dataset, ischemia was only present in 6% of PVC-positive patients and 2% of our series, indicating other pathophysiological factors are operative. Combined with the lack of evidence to support an association between PVC count and MI, findings seem to suggest that exercise and recovery PVCs are not necessarily related to myocardial ischemia.^9^

The relationship between PVCs recorded during rest and exercise remains to be further investigated. Beckerman et al^37^ studied 5754 male veterans referred for exercise testing and found that a combination of exercise-induced ventricular arrhythmias and resting PVCs carried a higher risk than exercise-induced ventricular arrhythmias alone (HR 1.6 versus HR 2.7). Although the authors did not precisely quantify the PVC rate at rest or during exercise as in our work, their findings may suggest complementarity mechanisms in modulating risk. Results from our sensitivity analyses showed that the risk associated with exercise-induced PVCs were independent from resting PVC burden, which may support this hypothesis. However, the resting ECGs (recorded preexercise) in this work were of very short duration (15 s) and therefore likely to have provided very limited sensitivity to detect PVCs because their occurrence is highly variable.

### Limitations

Our study benefited from several important advantages, including access to the largest and most detailed dataset of PVC activity from a population-based cohort without known cardiovascular disease, combined with long-term follow-up. Nevertheless, several limitations, apart from the limited sensitivity to detect myocardial ischemia, need to be acknowledged. First, there is a “healthy volunteer” selection bias in the UK Biobank with the participants being older and healthier than the general population. Furthermore, although the codes used in this study to determine the presence or absence of clinical conditions were carefully selected, we could not rule out misclassification of diagnoses or inaccuracies related to incorrect diagnoses. Second, participants performed submaximal exercise only and the cause of the PVCs may be different than in other studies where exercise was performed at maximal workload. However, we could already observe different event rates between PVC burdens, which might indicate stronger association with greater intensity exercise. Third, as discussed above, signs of exercise-induced myocardial ischemia may have remained undetected because our data were limited to single-lead ECG measurements. We therefore cannot fully rule out that PVCs were driven by ischemia. However, we believe this would be unlikely because it does not seem to be fully compatible with the observed lack of association between PVC burden and MI. Fourth, the study cohort is predominantly (>90%) of White British ancestry, which may limit the generalizability of our findings in underrepresented ethnicities, although the study is not likely to be flawed by an important comorbidity burden. Last, cardiac imaging and NT-proBNP data were only available in a small subset of our study cohort. Furthermore, imaging data were acquired several years after the exercise test and are therefore more likely to capture the effect of disease progression rather than the actual situation at the time of the test. Further evaluation is required to investigate the mechanisms underlying exercise-induced PVCs.

### Clinical Perspectives

In recent years, there has been a widespread use of wearable cardiac rhythm monitors, many of which are used during (submaximal) exercise. This has enabled the detection of PVCs in asymptomatic individuals at a larger scale and is expected to result in more consultation requests from concerned patients whose monitors detect PVCs. High PVC counts during submaximal exercise and recovery predict MI/HF/LTVA and all-cause mortality independent of standard cardiovascular risk factors in this middle-aged and older population. Clinicians need to be aware of the measurable prognostic significance of these PVCs to optimally assess and manage cardiovascular risk in these otherwise asymptomatic individuals to prevent life-threatening events.

### Conclusions

High PVC counts during exercise and recovery are associated with MI/HF/LTVA and all-cause mortality independent of standard cardiovascular risk factors. In addition, there is an incremental risk of MI/HF/LTVA for increasing PVC count, and complex PVCs have additional prognostic significance. This has major implications for risk stratification, screening, and interpretation of wearable ECG rhythm readouts in the middle-aged and older population and should be investigated in younger individuals.

## ARTICLE INFORMATION

### Sources of Funding

This study was supported by the MRC grant MR/N025083/1. Dr Lambiase is supported by University College Lundon/University College London Hospitals (UCL/UCLH) Biomedicine National Institute for Health and Care Research (NIHR). This work acknowledges the support of the National Institute for Health and Care Research Barts Biomedical Research Centre (NIHR203330); a delivery partnership of Barts Health National Health Service (NHS) Trust, Queen Mary University of London, St George’s University Hospitals NHS Foundation Trust, and St George’s University of London. Dr Ramírez acknowledges the “María Zambrano” fellowship support from the European Union-NextGenerationEU. Drs Doherty and van Duijvenboden also acknowledge support from the Wellcome Trust (223100/Z/21/Z). Dr Petersen acknowledges the British Heart Foundation for funding the manual analysis to create a cardiovascular magnetic resonance imaging reference standard for the UK Biobank imaging resource in 5000 cardiovascular magnetic resonance scans (www.bhf.org.uk; PG/14/89/31194). Barts Charity (G-002346) contributed to fees required to access UK Biobank data (access application #2964).

### Disclosures

Dr Petersen provides Consultancy to Circle Cardiovascular Imaging Inc., Calgary, Alberta, Canada. The remaining authors report no conflict.

### Supplemental Material

Tables S1–S16

Figures S1–S3

## Supplementary Material
