# Supplementary material for: Prognostic Significance of Different Ventricular Ectopic Burdens During Submaximal Exercise in Asymptomatic UK Biobank Subjects
Source: Circulation. 2023 Oct 19;148(24):1932–44. doi: 10.1161/CIRCULATIONAHA.123.064633 (PMC10712993; doi:10.1161/CIRCULATIONAHA.123.064633)
Supplement: Supplementary file 1 [file cir-148-1932-s001.pdf]

Figure S1 ECG QRS detection & classification

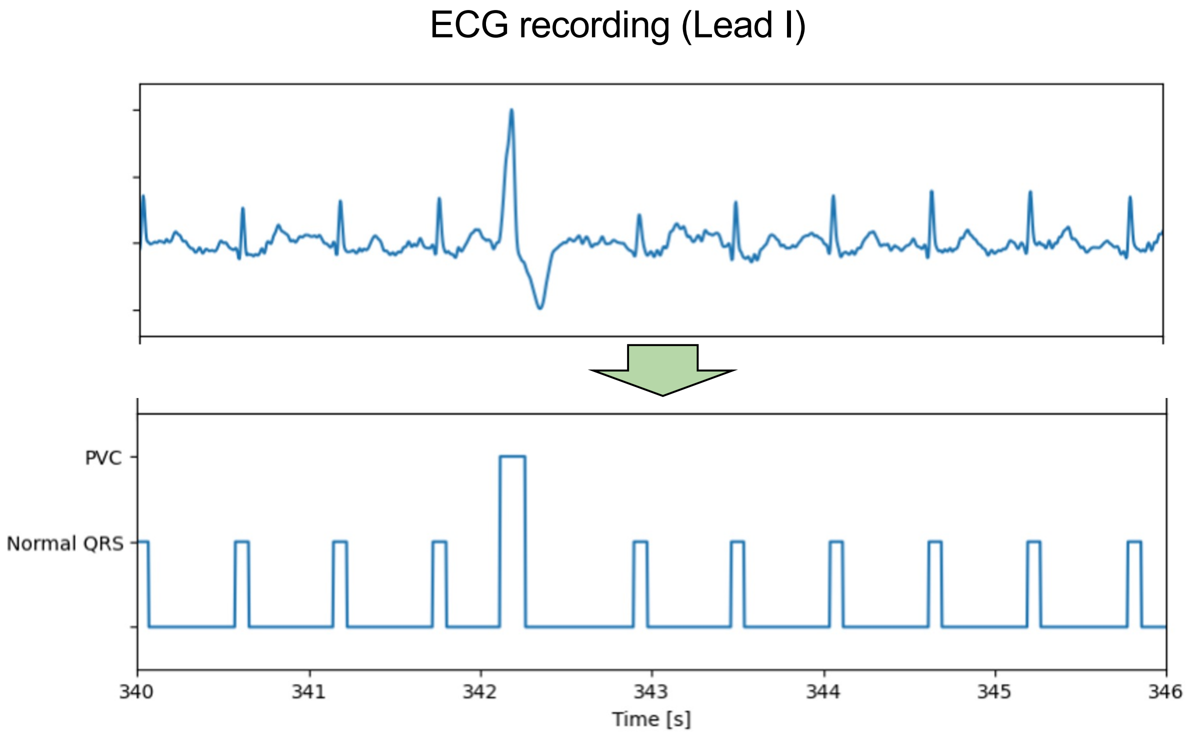

Figure S2 Distributions of PVC Counts

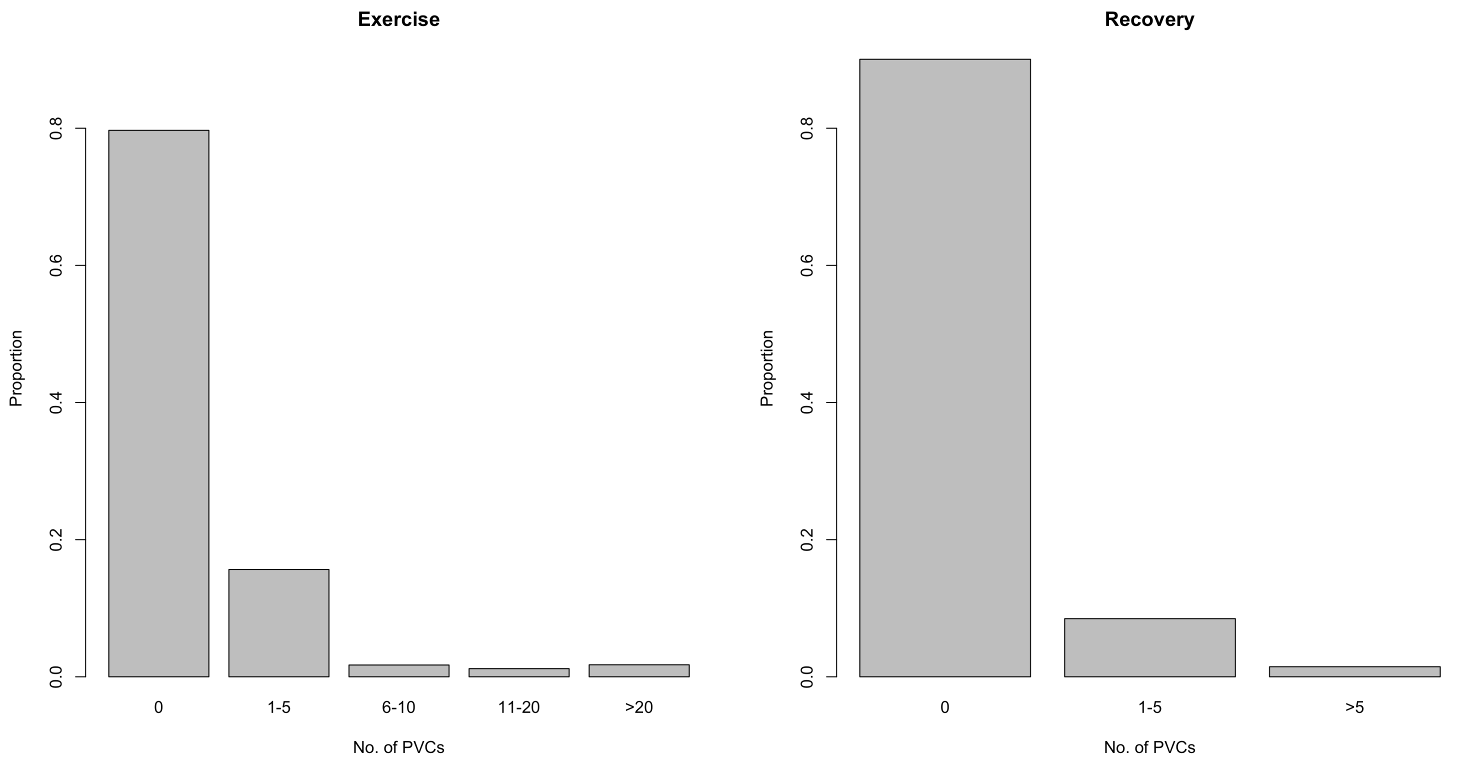

Figure S3: Adjusted Hazard Ratios of MI/HF/LTVA Events According to Presence of Different PVC Patterns

	Participants	Events
**High-grade PVCs**		
Exercise [a]	1560	146 (9.4%)
Recovery [b]	727	64 (8.8%)
**PVC repeating patterns [a + b]**		
1 couplet	85	12 (14.1%)
≥2 couplets	82	12 (14.6%)
≥3 consecutive PVCs	10	4 (40%)
Bigeminy	57	12 (21.1%)

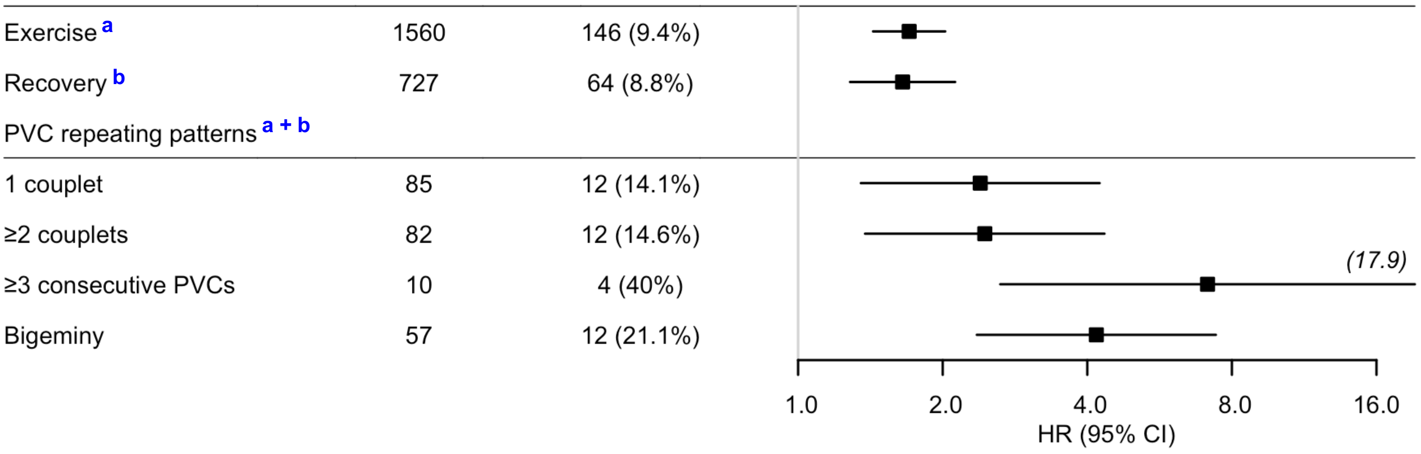

Hazard ratios were adjusted for clinical and exercise test variables and were all statistically significant ($P < 0.05$). Clinical variables: age, sex, diabetes, hypertension, beta-blocker medication, LDL and HDL cholesterol, triglycerides, body mass index, QRS duration, QTc interval, ST depression (>0.1 mV), and exercise ECG variables: [a]Total number of beats during exercise, and heart rate exercise; [b]Total number of beats during recovery, and heart rate recovery. HRs were referenced against participants without PVCs during exercise (*a*), recovery (*b*), or both (*a* + *b*). PVC: premature ventricular complex. HR hazard ratio, CI confidence interval.
